# Compare HIV/syphilis infections between age groups and explore associated factors of HIV/syphilis co-infections among men who have sex with men in Shenzhen, China, from 2009 to 2017

**DOI:** 10.1371/journal.pone.0223377

**Published:** 2019-10-03

**Authors:** Rong-Xing Weng, Fu-Chang Hong, Wei-Ye Yu, Yu-Mao Cai

**Affiliations:** Department of STD Control and Prevention, Shenzhen Center for Chronic Disease Control, Shenzhen, Guangdong Province, China; The Chinese University of Hong Kong, HONG KONG

## Abstract

The aim of this study is to assess the HIV/syphilis epidemic among men who have sex with men (MSM) aged <50 years and ≥50 years in Shenzhen, and explore the associated factors of HIV/syphilis co-infections among MSM in Shenzhen, in order to help prevention and intervention programs determine their target sub-group. A serial cross-sectional study was conducted on MSM in Shenzhen city, China from 2009 to 2017. A questionnaire was used to collect demographic characteristics, history of HIV testing, history of blood donation and sexual behaviors. 5 ml of venous blood were collected for syphilis and HIV tests. The overall prevalence of HIV, syphilis, HIV/syphilis co-infection was 9.40%, 18.97%, and 4.91%, respectively. The prevalence of HIV (15.26%), syphilis (27.71%), HIV/syphilis co-infection (9.24%) in aged ≥50 years MSM was significantly higher than aged <50 years MSM (9.15%, 18.59% and 4.72%, respectively). The following factors were found to be significantly associated with HIV/syphilis co-infections (***P***<0.05): age≥50 years (OR = 1.78, 95% CI = 1.10–2.87), high school or lower (OR = 1.49, 95% CI = 1.10–2.01), monthly income ≤436.2 USD (OR = 1.74, 95% CI = 1.25–2.42), monthly income 436.4–727.2 USD (OR = 1.46, 95% CI = 1.05–2.03), ≥2 anal sex partners in the past 6 months (OR = 1.59, 95% CI = 1.02–2.49), ≥2 oral sex partners in the past 6 months (OR = 1.60, 95% CI = 1.08–2.36), inconsistent condom use during anal sex in the past 6 months (OR = 1.50, 95% CI = 1.11–2.03). We found that aged <50 years and ≥50 years MSM in Shenzhen had a high prevalence of HIV/syphilis infection in a period from 2009 to 2017. Age-specific sexually transmitted diseases education, prevention, and intervention programs for aged ≥50 years MSM should be implemented urgently and integrated interventions of both HIV and syphilis infections on MSM are needed in the future.

## Introduction

Sexually transmitted diseases (STDs) such as Human Immunodeficiency Virus (HIV) infection/Acquired Immune Deficiency Syndrome (AIDS) and syphilis have become major public health problems worldwide. According to the World Health Organization (WHO), there are 376 million new sexually transmitted infections (STIs) worldwide with syphilis, chlamydia, trichomoniasis, and gonorrhea among women and men aged 15–49 years old in 2016, and about 37.9 million people are living with HIV at the end of 2018 [1, 2]. In past decades, the waves of HIV epidemic in China were caused by injection drug use and unsafe plasma donation, and the unprotected sex from Men Who Have Sex with Men (MSM) may be the cause of the next wave [3–5]. The burden of HIV-infection/AIDS was great in China, with 758,610 reported cases in 2017 [6]. It is found that 25.5% of the newly-discovered HIV-infected cases were transmitted via homosexual interactions, which shows a rise of the HIV epidemic in the MSM population from 2013 [6, 7]. Syphilis epidemic was also very serious in China, with 534,622 reported cases in 2017 [6]. According to a previous Meta-Analysis, the pooled prevalence of HIV and syphilis infection among MSM was high in China (6.5% and 11.2%, respectively) [8].

Several studies in China reported a similar prevalence of HIV/syphilis co-infections among MSM, ranging from 1.5% to 3.3% [7, 9–11]. A higher rate of asymptomatic primary syphilis and a more rapid infection progression to secondary syphilis was found in HIV infected patients from a previous study [12]. Also, untreated asymptomatic primary syphilis progress into secondary and tertiary stages which could lead to irreversible neurological or cardiovascular complications [13]. Therefore, understanding the HIV/syphilis co-infections in MSM is important.

Associated factors of HIV/syphilis co-infection among MSM were reported in several prior studies. Das et al. found that MSM with older age, unprotected anal intercourse in past six months, STD symptoms in the past year, incorrect knowledge about routes of transmission of HIV, accessing to HIV preventive services, and accessing to HIV/STI counseling/testing services had a higher risk of co-infection [10]. Polansky et al. reported that frequent unprotected anal intercourse with casual sex partners, limited partner notification, and inadequate adherence to antiretroviral therapy were significant positive predictors of having co-infection [14]. Diaz et al. suggested that the associated factors of HIV/syphilis co-infection among MSM in Spain were reporting exclusively anal intercourse, having STI diagnosis history, and having sex with casual or several types of partners [15]. Hernandez et al. found that MSM with older age, having multiple partners, and those who were forced to have sex were more likely to have co-infection [16].

In China, the aging population has grown significantly and is considered to keep increasing [17]. According to the world population report in 2017, the older population in China accounted for the largest proportion in the world [18]. This older population was proved to be more vulnerable to sexually transmitted infections and HIV in several studies and one possible reason for the higher vulnerability is the lack of sexual health knowledge in the older population [19–22]. The increase of STIs/HIV-infected cases among people aged 50 and above is becoming the new pattern of HIV epidemic in China [23, 24]. A previous study showed that an increasing proportion of HIV case report attributed to people aged 50 and above in Chongqing, China, from 2.2% in 2005 to 44.0% in 2015 [25] and the proportion of HIV case report attributed to people aged 50–59 years also became larger, increasing from 1.6% in 2005 to 15.9% in 2015 [25]. Another study reported an increasing proportion of HIV cases in China attributed to people aged 50–64 years old, from 6.1% in 2006 to 11.4% in 2009, so it is worth to consider both people aged 50–64 and ≥ 65 [26]. It was reported that those older MSM population (aged 50 and above) was found to have higher HIV prevalence than t their peers but not identified as MSM in Shanghai city, China [27]. Therefore, the older MSM population in China should be targeted in the STIs/HIV prevention and intervention programs.

Shenzhen city is located in the southeast of mainland China and is neighboring Hong Kong. As the first Special Economic Zone in China, Shenzhen is one of the fastest growing cities in China with a 22.4% annual average growth rate of gross domestic product (GDP) from 0.2 billion GDP in 1979 to 2249.0 billion GDP in 2017 [28]. According to the sixth national census in China in 2010, 7.5% of the total population in Shenzhen was people aged 50 and above [29] and it was estimated that there were around 100,000 MSM in Shenzhen [30]. Previous studies in Shenzhen showed that the prevalence of HIV and syphilis infection among MSM in Shenzhen was higher than the national average [31]. In recent years in Shenzhen, the prevalence of HIV infection among MSM has increased significantly (12.6% in 2016), and the prevalence of MSM syphilis infection has increased slowly but remain high [31, 32].

Given the burden of HIV, syphilis, and HIV/syphilis co-infections among MSM population, the increase of aging population, and the higher HIV prevalence in the older MSM population, the aim of this study is to assess the HIV/syphilis epidemic among MSM aged < 50 years and ≥ 50 years in Shenzhen and explore the associated factors of HIV/syphilis co-infections among MSM in Shenzhen, in order to help prevention and intervention programs determine their target sub-groups.

## Materials and methods

### Subjects

The current study was a serial cross-sectional study on MSM from 2009 to 2017 in Shenzhen city, Guangdong Province, China. The rainbow clinic in Shenzhen Center for Chronic Disease Control was one of the largest STDs clinics and MSM attending this clinic were from many different areas throughout the city, thus the MSM population in Shenzhen may be represented by MSM in this rainbow clinic to some extent. Convenience sampling method based on this rainbow clinic was used to recruit MSM in this study. The criteria of eligible participants were: (1) age ≥ 18 years; (2) men who had anal or oral sex with another man in the last 12 months; (3) willing to participate and cooperate in the study and provide informed consent. Individuals who had participated in the study were excluded for avoiding duplicate analysis and measurement. Free treatment was provided for all newly discovered syphilis cases and referral services were provided for HIV-positive cases. To ensure confidentiality, respondents’ questionnaires, biological specimens, and test results were anonymized by assigning unique survey identification numbers. Written informed consent was obtained from each participant.

### Behavioral measures

Intensive training and detailed protocols were provided to interviewers in Shenzhen Center for Chronic Disease Control. After confirming the eligibility of the individuals and acquiring their written informed consent, the trained interviewers conducted a one-on-one interview using the same structured questionnaire to obtain participants’ information in a private room in the rainbow clinic. The questionnaire included demographic characteristics (e.g. age, education level, and occupation), history of HIV testing, history of blood donation and sexual behaviors (e.g. condom use, number and types of partners). Contact information (e.g. mobile phone number, email address or home phone number) was also obtained to inform biological test results and referral services if necessary.

### Biological measures

After the interview, 5 ml of venous blood were collected for syphilis and HIV tests. The screening of treponema pallidum antibody was conducted using the tolulized red unheated serum test (TRUST; Rongsheng Biotech Inc, Shanghai, China). Positive screening results from TRUST were verified by the treponema pallidum particle agglutination test (TPPA; Fujirebio Inc. Japan). The specimen with positive results in both TRUST and TPPA could be diagnosed as syphilis infection. HIV screening was performed by using enzyme-linked immunoassays (ELISAs; Wantai BioPharm Inc, Beijing, China). The ELISA-positive participants were confirmed by the Western Blot (WB) test (WB; MP Biomedicals Asia Pacific Pte Ltd, Singapore).

### Statistical analysis

Data were double entered and cleaned using the Epi Data software (Epi Data for Windows; The Epi Data Association Odense, Denmark). The data of continuous variables and categorical variables were presented as mean ± SD and frequency (%), respectively. The ***χ***^***2***^ test was used to explore the differences between aged < 50 years MSM and those ≥ 50 years and Linear-by-Linear Association test was used to explore the prevalence trends of HIV, syphilis and HIV/syphilis co-infection. Univariate logistic regression analysis was used to obtain crude odds ratios (OR) and their 95% CIs, variables with ***P***<0.20 in the univariate logistic regression analysis were included in the multivariate logistic regression analysis. We used a forward stepwise procedure in the multivariate logistic regression analysis to obtain adjusted odds ratios (AOR) and their 95% CIs. We adopted a multivariable logistic regression model, defining HIV/syphilis co-infections as the dependent variable and age, education level, monthly income, number of anal sex partners in the past 6 months, number of oral sex partners in the past 6 months, number of female partners in the past 6 months, condom use during anal sex in the past 6 months, condom use during last anal sex, history of blood donation as the independent variables. All tests were two-tailed, with statistical significance defined as a P<0.05. All data analysis was performed using Statistical Package for Social Sciences (SPSS) version 21.0 software (SPSS Inc., Chicago, IL, USA).

### Ethics approval

Ethical review and approval were obtained from the ethical review committee of Shenzhen Center for Chronic Disease Control (approval number 2008NL031).

## Results

### Background characteristics

Background characteristics of participants were shown in Table 1. From 2009 to 2017, totally 5,966 subjects were recruited. The age range of the participants was from 15 to 73, with a mean of 31.2 ± 8.6 years. More than 70% of participants did not have HIV test history and more than 40% of them perceived themselves as bisexual. More aged ≥ 50 years MSM (77.91%) received high school or lower education than aged < 50 years MSM (60.14%) (*P*<0.001). More aged ≥ 50 years MSM (79.92%) are currently married than those aged < 50 years (23.82%) (*P*<0.001). More aged ≥ 50 years MSM (33.34%) were unemployed or retired than aged < 50 years MSM (8.31%) (*P*<0.001). More aged ≥ 50 years MSM (51.00%) had low monthly income (≤ 436.2 USD) than aged < 50 years MSM (29.44%) (*P*<0.001). Aged ≥ 50 years MSM were less likely to donate blood than aged < 50 years MSM (6.07% versus 17.48%, *P*<0.001).

**Table 1:** Background characteristics of participants.

Variables	<50 years	≥50 years	*P* value^d^
	N^a^ (%)^b^	N^c^ (%)^b^	
Junior college or higher			
No	3437 (60.14%)	194 (77.91%)	<0.001**
Yes	2278 (39.86%)	55 (22.09%)	
Currently married			
No	4355 (76.18%)	50 (20.08%)	<0.001**
Yes	1362 (23.82%)	199 (79.92%)	
Currently occupation			
Money boy	159 (2.78%)	3 (1.20%)	<0.001**
Unemployed/ retired	475 (8.31%)	83 (33.34%)	
Others	5083 (88.91%)	163 (65.46%)	
Monthly income, RMB			
≤2999 ≈ 436.2 USD	1683 (29.44%)	127 (51.00%)	<0.001**
3000–4999 ≈ 436.4–727.2 USD	1749 (30.59%)	67 (26.91%)	
≥5000 ≈ 727.3 USD	2285 (39.97%)	55 (22.09%)	
History of HIV testing			
No	1403 (24.62%)	69 (27.82%)	0.252
Yes	4296 (75.38%)	179 (72.18%)	
History of blood donation			
No	4712 (82.52%)	232 (93.93%)	<0.001**
Yes	998 (17.48%)	15 (6.07%)	
Self-perceived sexual orientation			
Homosexual	3293 (57.60%)	128 (51.61%)	0.062
Bisexual	2424 (42.40%)	120 (48.39%)	
Heterosexual	52 (0.91%)	2 (0.81%)	

^a^N for each subgroup may not equal to the number of 5717, because of missing data.

^b^%: Constituent ratio.

^c^N for each subgroup may not equal to the number of 249, because of missing data.

^d^χ^2^ Test.

**P<0.001.

### Sexual behaviors

Sexual behaviors of participants in the past six months were shown in Table 2. Aged < 50 years MSM (7.35%) are more likely to have at least 2 female sexual partners than those aged ≥ 50 years MSM (3.73%) (*P*<0.05). Similarly, those MSM aged < 50 years (68.07%) are more likely to have at least 2 anal sexual partners (male) than aged ≥ 50 years MSM (61.38%) (*P*<0.05). Those aged ≥ 50 years MSM (13.56%) are less likely to use condom consistently during sex with female in the past six months than aged < 50 years MSM (33.89%) (*P*<0.05). More aged ≥ 50 years MSM (85.19%) did not use condom during last sex with female than aged <50 years MSM (54.93%) (*P*<0.001). Similarly, more aged ≥ 50 years MSM (37.56%) did not use condom during last anal sex with male than aged < 50 years MSM (28.93%) (*P*<0.05). The proportion of inconsistent condom use during anal and oral sex in the past six months was high in the whole population (58.63% and 96.72%, respectively), and most of them did not use condom during last oral sex. There was no significant difference in oral sexual behaviors with male between the two groups.

**Table 2:** Sexual behaviors, HIV and syphilis infection of participants.

Variable	<50 years	≥50 years	*P* values^d^
	N^a^ (%)^b^	N^c^ (%)^b^	
**Sexual behavior with women**			
Number of female partners in the past 6 months			
0–1	5281 (92.65%)	232 (96.27%)	0.033*
≥2	420 (7.35%)	9 (3.73%)	
Condom use during sex in the past 6 months			
Inconsistent	1028 (66.11%)	51 (86.44%)	0.001*
Consistent	527 (33.89%)	8 (13.56%)	
Condom use during last sex			
No	629 (54.93%)	46 (85.19%)	<0.001**
Yes	516 (45.07%)	8 (14.81%)	
**Sexual behavior with men**			
Number of anal sex partners in the past 6 months			0.028*
0–1	1825 (31.93%)	95 (38.62%)	
≥2	3891 (68.07%)	151 (61.38%)	
Condom use during anal sex in the past 6 months			
Inconsistent	3058 (58.40%)	137 (64.32%)	0.086
Consistent	2178 (41.60%)	76 (35.68%)	
Condom use during last anal sex			
No	1508 (28.93%)	80 (37.56%)	0.007*
Yes	3705 (71.07%)	133 (62.44%)	
Number of oral sex partners in the past 6 months			
0–1	2028 (35.65%)	92 (37.55%)	0.544
≥2	3660 (64.35%)	153 (62.45%)	
Condom use during oral sex in the past 6 months			
Inconsistent	4809 (96.78%)	204 (95.33%)	0.243
Consistent	160 (3.22%)	10 (4.67%)	
Condom use during last oral sex			
No	4625 (93.11%)	200 (93.46%)	0.846
Yes	342 (6.89%)	14 (6.54%)	
HIV infection			
No	5194 (90.85%)	211 (84.74%)	0.001*
Yes	523 (9.15%)	38 (15.26%)	
Syphilis infection			
No	4654 (81.41%)	180 (72.29%)	<0.001**
Yes	1063 (18.59%)	69 (27.71%)	
HIV/syphilis co-infection			
No	5447 (95.28%)	226 (90.76%)	0.001*
Yes	270 (4.72%)	23 (9.24%)	

^a^N for each subgroup may not equal to the number of 5717, because of missing data.

^b^%: Constituent ratio.

^c^N for each subgroup may not equal to the number of 249, because of missing data.

^d^χ^2^ Test.

*P<0.05

**P<0.001.

### Infections of HIV and syphilis

The overall prevalence of HIV, syphilis, HIV/syphilis co-infection was 9.40%, 18.97% and 4.91%, respectively (Table 2). The prevalence of HIV (15.26%), syphilis (27.71%), HIV/syphilis co-infection (9.24%) in aged ≥ 50 years MSM was significantly higher than those aged < 50 years (9.15%, 18.59%, and 4.72%, respectively) (Table 2). Trends of HIV, syphilis, and HIV/syphilis co-infection prevalence were shown in Table 3. A decreasing trend of syphilis infection was found in both MSM aged < 50 and ≥ 50. An increasing trend of HIV prevalence was found in MSM aged < 50. For others, the prevalence fluctuated throughout these nine years.

**Table 3:** Trends of HIV, syphilis, and HIV/syphilis co-infection prevalence.

Infection	2009	2010	2011	2012	2013	2014	2015	2016	2017	*P* Values^a^
Syphilis N (%)										
Age<50	207 (23.10%)	247 (19.92%)	130 (17.64%)	87 (18.20%)	114 (17.22%)	102 (17.86%)	86 (19.07%)	54 (14.71%)	36 (11.43%)	<0.001**
Age≥50	9 (69.23%)	12 (37.50%)	6 (24.00%)	5 (20.83%)	11 (40.74%)	9 (29.03%)	6 (17.65%)	7 (22.58%)	4 (12.50%)	0.001*
HIV N (%)										
Age<50	80 (8.93%)	90 (7.26%)	52 (7.06%)	46 (9.62%)	63 (9.52%)	76 (13.31%)	41 (9.09%)	41 (11.17%)	34 (10.79%)	0.001*
Age≥50	2 (15.38%)	6 (18.75%)	2 (8.00%)	3 (12.50%)	5 (18.52%)	6 (19.35%)	3 (8.82%)	5 (16.13%)	6 (18.75%)	0.830
HIV/Syphilis co-infection N (%)										
Age<50	47 (5.25%)	47 (3.79%)	27 (3.66%)	23 (4.81%)	29 (4.38%)	42 (7.36%)	22 (4.88%)	16 (4.36%)	17 (5.40%)	0.186
Age≥50	2 (15.38%)	4 (12.50%)	2 (8.00%)	2 (8.33%)	5 (18.52%)	1 (3.23%)	1 (2.94%)	2 (6.45%)	4 (12.50%)	0.377

^a^Linear-by-Linear Association test.

*P<0.05

**P<0.001.

### Associated factors of HIV/syphilis co-infections

The results (Table 4) from the multivariable logistic regression model suggested that the following factors were found to be significantly associated with HIV/syphilis co-infections (***P***<0.05): age ≥ 50 years (OR = 1.78, 95% CI = 1.10–2.87), high school or lower (OR = 1.49, 95% CI = 1.10–2.01), monthly income ≤ 436.2 USD (OR = 1.74, 95% CI = 1.25–2.42), monthly income 436.4–727.2 USD (OR = 1.46, 95% CI = 1.05–2.03), ≥ 2 anal sex partners in the past 6 months (OR = 1.59, 95% CI = 1.02–2.49), ≥ 2 oral sex partners in the past 6 months (OR = 1.60, 95% CI = 1.08–2.36), inconsistent condom use during anal sex in the past 6 months (OR = 1.50, 95% CI = 1.11–2.03).

**Table 4:** Multivariable logistic regression analysis of associated factors of HIV/syphilis co-infections.

Independent variables	Adjusted OR (95% CI)	*P* Values
Age≥50 years		
Yes	1.78 (1.10, 2.87)	0.018*
No	Reference	
Junior college or higher		
Yes	Reference	
No	1.49 (1.10, 2.01)	0.010*
Monthly income, RMB		
≤2999 ≈ 436.2 USD	1.74 (1.25, 2.42)	0.001*
3000–4999 ≈ 436.4–727.2 USD	1.46 (1.05, 2.03)	0.023*
≥5000 ≈ 727.3 USD	Reference	
Number of anal sex partners in the past 6 months		
0–1	Reference	0.041*
≥2	1.59 (1.02, 2.49)	
Number of oral sex partners in the past 6 months		
0–1	Reference	0.019*
≥2	1.60 (1.08, 2.36)	
Number of female partners in the past 6 months		
0–1	Reference	0.160
≥2	0.66 (0.37, 1.18)	
Condom use during anal sex in the past 6 months		
Inconsistent	1.50 (1.11, 2.03)	
Consistent	Reference	0.009*
Condom use during last anal sex		
No	1.06 (0.79, 1.42)	
Yes	Reference	0.703
History of blood donation		
No	1.31 (0.91, 1.89)	0.145
Yes	Reference	

Abbreviations: OR, odds ratio; CI, confidence interval.

*P<0.05

## Discussion

In the current study, HIV prevalence among MSM aged ≥ 50 years was higher than those aged < 50 years (15.3% versus 9.2%). The prevalence among older MSM, (15.3%) was higher than the pooled prevalence among older MSM in a previous meta-analysis (11.6%) [33], and was higher than the average prevalence among older MSM in a nationwide cross-sectional survey (7.6%) [34]. Similarly, in the present study, syphilis prevalence among MSM aged ≥ 50 years was higher than MSM aged <50 years (27.7% versus 18.6%), and both were much higher than the prevalence among older MSM in the previous study in Shanghai (12.4%) [27]. The above phenomenon may be explained by several reasons. Firstly, older MSM were exposed to HIV source with longer period [33]. Secondly, after retirement, older males were likely to engage in commercial sexual activities [19], which led to the rise of HIV/syphilis prevalence in the population of China’s older males [7, 35]. A high rate of inconsistent condom use at commercial sex was reported in several studies [36–38], and those with inconsistent condom use at commercial sex had a higher risk of the HIV/syphilis infection than those with consistent condom use [36, 39]. Besides, selling sex was identified as a risk factor of the HIV/syphilis infection because of the high frequency of unprotected anal intercourse and multiple sexual partners [40, 41]. Lastly, many older people held their perceptions about HIV. Some of them believed they were at low risk of HIV infection [42, 43]. What was worse, some of them were not concerned about HIV, because they believed that the latent period to AIDS could be long, and they might be already dead due to other diseases [33]. In addition, we inferred that, due to the accessibility and the improvement of HIV treatment, older adults might consider AIDS as a chronic disease. These perceptions may be the reasons for the higher HIV prevalence in older MSM.

In the present study, the prevalence of HIV/syphilis co-infections (9.2%) among older MSM was much higher than several previous studies (2.6% in China and 4.8% in Ecuador) [10, 16], which suggests that integrated interventions and screening for both HIV and syphilis infections on older MSM could be considered. A possible reason of the higher prevalence of co-infections in our study was that many rural-to-urban migrants looked for economic opportunities in China, and this population was found to have limited education, lower income, and more vulnerable to STIs [44, 45]. There are more migrant workers in Shenzhen than the national level. In our study, the prevalence of HIV/syphilis co-infection (9.2%) in aged ≥ 50 years MSM was significantly higher than aged < 50 years MSM (4.7%) and older MSM had a higher risk of HIV/syphilis co-infections than younger MSM, which was consistent with previous studies [10, 46]. This high risk that older MSM face may be explained by several reasons. Firstly, many older adults lacked STDs related-knowledge, which led to unawareness of the inherent riskiness in unprotected sexual behaviors [47]. Secondly, older age group was not targeted in most STDs education programs [48], so many of older MSM feel nervous to participate in STDs-related education, prevention and intervention programs which were surrounded by young people. Lastly, discussion about sexual health or sexual safety between physicians and older adults was rare [47], due to the culture-related embarrassment to talk about sensitive, private content from both sides. Without obtaining professional health-maintenance information from their trusted doctors, these older MSM would not modify their risky sexual behaviors [49]. Given the above situation, age-specific STDs education, prevention, and intervention programs for MSM aged ≥ 50 years should be implemented. A previous study indicated that the difference of informational and intervention needs between younger and older adults was great, and the risk factors of HIV infection for older men were different from those of younger men [49]. It suggested that some effective STDs interventions on younger MSM might not fit their older counterparts, and age-specific risk factors of STDs among older MSM should be considered [49].

Although there was no significant difference in most unprotected sexual behaviors with men between MSM aged ≥ 50 years and < 50 years, the proportion of these risky behaviors in older MSM was still appeared to be higher, which was consistent to the pooled prevalence in a previous meta-analysis [33]. Compared to MSM aged < 50 years, older MSM were more likely to engage in condomless sex with females in the current study, which may act as a bridge in the HIV and other STDs spread between the high-risk group and the general population [50].

The current study found that participants with lower education had a higher risk of HIV/syphilis co-infection than those with higher education, which was consistent with findings in other studies [46, 51]. However, education level is not the only social- demographic factor of HIV/syphilis co-infections. We found that MSM with lower monthly income had a higher risk of HIV/syphilis co-infections, which was consistent with a previous study [52]. Therefore, MSM with lower education or lower monthly income should be targeted with high priority in future intervention. In addition, stigma and discrimination against homosexuality among the general population in China is persistent, which may contribute to the STDs spread among MSM [53]. Therefore, education on the general population to reduce stigma and discrimination against homosexuality is also necessary.

This study suggested that MSM with more anal/oral sex partners and inconsistent condom use during anal sex had a higher risk of HIV/syphilis co-infection than those with fewer sex partners and consistent condom use, which was in accordance with findings in previous studies [14, 16, 54, 55]. More sex partners may indicate more opportunities to engage in unprotected sexual behaviors, so partner notifications should be further promoted in this population. Furthermore, disclosure of HIV status and enhanced partner communication skills may help reduce HIV risk and unprotected sexual behavior [49, 56, 57], so related education and prevention programs should be considered.

In the current study, several potential limitations should be acknowledged. Firstly, this study was conducted in a single city, which may affect the generalizability of the study. Secondly, social desirability bias and recall bias may exist. One-on-one interviews were conducted by well-trained interviewers according to an identical protocol to minimize the bias. Thirdly, the recruitment rate could not be obtained in our study because related data was not collected. Lastly, our findings were based on the convenience sampling, which may hinder the representativeness of the present study. Besides, this recruitment method may lead to selection bias. The rainbow clinic in our study is more likely to act as a voluntary counseling and testing (VCT) site and MSM come here mainly for counseling and routine testing. MSM who attending the clinic may be more sexually active or have more risky sexual behaviors, which may attribute to the higher HIV and syphilis prevalence. However, it is hard to recruit MSM using random sampling because of the stigma among MSM and the discrimination from the general population in China. Additionally, the data in the study were collected in the rainbow clinic in Shenzhen Center for Chronic Disease Control, which may cause bias since young MSM may prefer other community-based organizations for testing.

## Conclusions

This study indicated that both aged < 50 years and ≥ 50 years MSM in Shenzhen had a high prevalence of HIV/syphilis infection in a period from 2009 to 2017. Age-specific information on STDs education, prevention, and intervention programs for aged ≥ 50 years MSM should be implemented urgently. Our findings suggested age, education, monthly income, the number of anal/oral sex partners in the past 6 months, consistent condom use during anal sex in the past 6 months are associated factors of HIV/syphilis co-infections.

## Supporting information

S1 File Supporting Information-Survey questions in English. (DOC)

S2 File Supporting Information-Survey questions in Chinese. (DOC)
