# Supplementary material for: Compare HIV/syphilis infections between age groups and explore associated factors of HIV/syphilis co-infections among men who have sex with men in Shenzhen, China, from 2009 to 2017
Source: PLoS One. 2019 Oct 3;14(10):e0223377. doi: 10.1371/journal.pone.0223377 (PMC6776337; doi:10.1371/journal.pone.0223377)
Supplement: S1 File — (DOC) [file pone.0223377.s001.doc]

**MSM survey questionnaires**

Survey identification numbers:

| Age (year) |  | Marital status |  |
| --- | --- | --- | --- |
| Education level |  | Monthly income (RMB Yuan): ___ | |
| History of HIV testing | £Yes £No | Self-perceived sexual orientation | £Homosexuality  £Bisexuality  £Heterosexuality |
| History of blood donation | £Yes £No | Currently occupation |  |
| Contact information |  | | |
| Number of female partners in the past 6 months: ___ | | Condom use during sex in the past 6 months (with women):  £Consistent £Inconsistent | |
|  |  | Condom use during last sex: £Yes £No | |
| Number of anal sex partners in the past 6 months (with men): ___ | | Condom use during anal sex in the past 6 months (with men):  £Consistent £Inconsistent | |
|  |  | Condom use during last anal sex:  £Yes £No | |
| Number of oral sex partners in the past 6 months (with men): ___ | | Condom use during oral sex in the past 6 months (with men):  £Consistent £Inconsistent | |
|  |  | Condom use during last oral sex:  £Yes £No | |
|  | | | |
