# Supplementary material for: Compare HIV/syphilis infections between age groups and explore associated factors of HIV/syphilis co-infections among men who have sex with men in Shenzhen, China, from 2009 to 2017
Source: PLoS One. 2019 Oct 3;14(10):e0223377. doi: 10.1371/journal.pone.0223377 (PMC6776337; doi:10.1371/journal.pone.0223377)
Supplement: S2 File — (DOC) [file pone.0223377.s002.doc]

**MSM调查问卷**

编号：

| 年龄 |  | 婚姻 |  |
| --- | --- | --- | --- |
| 文化程度 |  | 月收入：______元 | |
| 既往HIV检测 | £是 £否 | 性取向 | £同 □双 □异 |
| 献血史 | □是 £否 | 职业 |  |
| 联系方式 |  | | |
| 最近半年，您和___个女性发生过性行为 | | *最近半年和女性性交时使用安全套： | |
|  |  | 最近一次性行为时是否使用安全套：□是 □否 | |
| 最近半年，您和____个男性发生过肛交 | | *最近半年和男性肛交时使用安全套： | |
|  |  | 最近一次肛交时是否使用安全套：□是 □否 | |
| 最近半年，您和_____个男性发生过口交 | | *最近半年和男性口交时使用安全套： | |
|  |  | 最近一次口交时是否使用安全套：□是 □否 | |
| *备注：安全套使用频率①从不或者有时用 ②每次用 | | | |
